# Strengthening the planning and design of Objective Structured Clinical Examinations

**DOI:** 10.4102/hsag.v29i0.2693

**Published:** 2024-08-07

**Authors:** Thandolwakhe Nyangeni, Wilma ten Ham-Baloyi, Dalena R.M. van Rooyen

**Affiliations:** 1 Department of Nursing Science, Faculty of Health Sciences, Nelson Mandela University, Gqeberha, South Africa; 2 Faculty of Health Sciences, Nelson Mandela University, Gqeberha, South Africa

**Keywords:** recommendations, Objective Structured Clinical Examinations, evidence-based, design, planning, strengthening

## Abstract

**Background:**

Although Objective Structured Clinical Examinations (OSCEs) offer innovative, objective, and fair methods of clinical assessment, their quality is compromised by poor planning and design.

**Aim:**

This study aimed to describe the development and present evidence-based recommendations on strengthening the planning and design of OSCEs for a South African public College of Nursing.

**Setting:**

A South African public College of Nursing.

**Methods:**

Recommendations were developed based on synthesising two sets of qualitative data. Set 1 included two main themes with each of the four sub-themes related to barriers and facilitators towards quality in OSCE designs from 14 nurse educator interviews and 15 external moderator reports. Set 2 included 12 quality measures to be adopted in the quality design of OSCEs derived from an integrative literature review. The draft recommendations were reviewed by eight experts to be finalised.

**Results:**

Seven recommendations were developed for strengthening OSCEs’ planning and design, related to: (1) policy framework, standard operating procedures and stakeholder code of conduct; (2) blueprinting and mapping of the OSCE content; (3) developing a bank of OSCE stations; (4) scoring rubric and standard-setting method selection; (5) examiners and standardised patients’ recruitment and training; (6) venue selection; and (7) station piloting.

**Conclusion:**

The seven developed recommendations can strengthen the quality of OSCEs in the South African public College of Nursing context.

**Contribution:**

The developed recommendations can assist nurse educators in planning and designing to conduct quality OSCEs following piloting and implementation.

## Introduction

Objective Structured Clinical Examinations (OSCEs) represent a dynamic assessment approach for formative and summative clinical evaluations. They entail a series of time-constrained stations where students undertake structured tasks while undergoing independent assessment by examiners within a secure setting (Castellani et al. 2020). Objective Structured Clinical Examinations promote evaluating affective, cognitive, and psychomotor learning domains on large student numbers within a short time using realistic clinical scenarios in a safe simulation laboratory environment (Castellani et al. 2020). In the affective domain, students’ attitudes, values, and professionalism are assessed, allowing educators to gauge their interpersonal skills, empathy, and ethical decision-making. Within the cognitive domain, OSCEs evaluate students’ knowledge, understanding, and critical thinking abilities related to clinical concepts, diagnostic reasoning, and treatment planning. Furthermore, in the psychomotor domain, students’ physical skills, procedural competency, and clinical techniques are scrutinised, providing insights into their proficiency in executing clinical tasks effectively. By encompassing these diverse domains, OSCEs offer a holistic approach to assessing students’ competency and readiness for clinical practice (Castellani et al. 2020). In an OSCE, students perform similar clinical tasks, and their performance is graded against a predetermined scoring rubric, thereby promoting uniformity, validity, and reliability of assessment (Cheema & Ali 2021). Objective Structured Clinical Examinations, as compared to traditional assessment methods such as mini clinical evaluations and case-based discussions, are innovative and provide for objectivity, standardisation and fairness and are, therefore, the preferred method for clinical assessment (Malau-Aduli et al. 2022).

While OSCEs have numerous benefits compared to traditional methods of clinical assessment, they require considerable resources, including time, personnel, and facilities. They are therefore expensive and require substantial preparation and planning (Bdair, Abuzaineh & Burqan 2019; Cheema & Ali 2021). Furthermore, maintaining consistent standardisation across multiple stations and examiners can be challenging, which may result in variations in scoring and evaluation. Despite efforts to simulate clinical scenarios, OSCEs may lack the complexity and realism of actual patient encounters, limiting their ability to assess clinical competence fully.

The timed, high-stakes nature of OSCEs can cause stress and anxiety in students, potentially impacting their performance and distorting assessment outcomes (Bdair et al. 2019).

Poorly planned and designed OSCEs and human errors may compromise accurate implementation of the examinations, leading to questionable credibility, uniformity, validity and reliability of results (John et al. 2021). Therefore, OSCE planning and design must be based on robust research evidence to strengthen their quality, credibility, and legal defensibility. Aligning OSCE design with international academic standards, and evidence-based recommendations could assist in this regard (John et al. 2021; Krusen & Rollins 2019).

Globally, evidence-based recommendations and guidelines for implementing OSCEs are available (Dewan, Khalil & Gupta 2024). However, these guidelines do not consider resource-constraint settings and are therefore not adaptable to the study context (a South African public College of Nursing). In the research context, the authors observed that the planning and design phase of OSCEs is often compromised, particularly among campuses with limited resources. For example, campuses lacking adequate spacing, trained examiners, and manikins struggled to plan and design real-life scenarios effectively. Additionally, at the time of the study, there were no existing evidence-based recommendations or guidelines to assist nurse educators in strengthening the planning and design of OSCEs, leading to inconsistencies in examinations. Therefore, the study aims to develop and present evidence-based recommendations (hereby referred to as ‘recommendations’) to strengthen the planning and design of OSCEs for a South African public College of Nursing.

## Research design and methods

A qualitative research design was employed, incorporating multiple methods such as individual semi-structured interviews with nurse educators and a document analysis of data extracted from external moderator reports. In addition, an integrative literature review summarising existing literature on best practices for managing the quality of OSCEs in health science education was conducted. The qualitative data from the interviews and document analysis, along with the findings from the integrative literature review, were synthesised to develop comprehensive recommendations for enhancing the design of OSCEs at a South African public College of Nursing. This article that forms part of a larger doctoral study that developed a best practice guideline managing quality OSCEs at a South African public College of Nursing, focusses on the planning and design of an OSCE.

### Setting

The study was conducted at a South African public College of Nursing consisting of five main and 19 satellite campuses. When the study was conducted (2021), the college had approximately 1500 enrolled students and 128 nurse educators employed to assist with offering a basic 4-year nursing diploma programme. For clinical examinations, OSCEs are the preferred method for summative clinical examinations at this college and count for 50% towards the final mark for the clinical subject or module (primary healthcare, psychiatric nursing, or midwifery). Objective Structured Clinical Examinations for the aforementioned subjects are conducted exclusively at the five main campuses, so the study included only these campuses. During an OSCE, conducted over 1 day simultaneously at each of the five campuses, approximately 350–400 students are examined, divided over 8–10 OSCE stations, with each station examining the same clinical skill in approximately 20–40 min. An examination is conducted independently by two examiners per station, using a pre-developed checklist, which is externally moderated by at least 1 of the 20 academics (nurse educators referred to as ‘external moderators’) employed at one of three independent universities.

### Methods

The methods used in the qualitative interviews, document analysis and integrative literature review are explained as follows.

#### Qualitative interviews

Fourteen nurse educators (*N* = 14), each with at least 2 years’ expertise in nursing education and who were involved in conducting OSCEs from the five campuses, were purposely selected to participate. Face-to-face, individual interviews were conducted and audio-recorded by the first author – a male nurse educator and master’s degree holder with experience in conducting qualitative interviews – to obtain interviewees’ experiences regarding quality OSCE management at the college under study. The majority (*n* = 13) of the 14 participating nurse educators were female, with ages ranging from 33 to 60 years, with between 3 and 30 years of work experience.

The first three interviews conducted with nurse educators from Campus Five served as the pilot study. As the first author was employed at this campus, pilot interviews were conducted by the third author – a female nurse educator with a PhD in Nursing Science – who was neither affiliated with the campus nor had a pre-established relationship with any participants. No changes were needed to the interview schedule and the data were included in the main study.

Interviews took between 60 and 90 min each and were conducted in a venue and at a time that were convenient for the participants. Data were collected during a 2-month period in 2019. Immediately after the interviews, the first author transcribed the recordings verbatim and coded the data with the assistance of an independent coder. The data were categorised into two themes with four sub-themes each, related to facilitators and barriers of quality OSCE designs, according to Tesch’s data analysis method. Tesch’s data analysis method involved systematically coding qualitative data by organising, categorising, and interpreting themes by the first author, with assistance of the co-coder to derive meaningful insights (Weyant 2022) (see Table 3 for the themes).

#### Document analysis

Following the completion of the interviews, the first author examined the documents. These documents consisted of reports provided by external moderators regarding the college’s OSCEs. The selection process involved picking a deliberate set of 30 extensively detailed reports from external moderators (*n* = 30). To extract information about comments related to the OSCEs’ planning and design, a data extraction tool developed by the first author was utilised (see Table 1 with an example).

**TABLE 1:** Document analysis’ data extraction tool.

Campus	Report number and page number(s)	Comments on OSCE planning and design
2	Report Two, page 67	Educate individuals on the significance of consistency in both marking and timekeeping.

OSCE, Objective Structured Clinical Examination.

The point of data saturation was achieved after scrutinising 15 of the external moderator reports (*n* = 15). The process of thematic analysis was conducted by the first author in collaboration with an independent coder. This involved thoroughly reviewing the extracted data, performing manual coding, and categorising the content into coherent topics. These topics were further organised into three overarching themes and their respective sub-themes. As a result of the large overlap with the data from qualitative interviews, the themes derived from the document analysis were merged with those of the qualitative interviews, resulting in two main themes with four sub-themes, each related to facilitators and barriers of quality OSCE planning and design (see Table 3).

#### Integrative literature review

An integrative literature review was conducted to summarise existing literature regarding best practices for managing the quality of OSCEs in health science education. Table 2 outlines the search strategy employed.

**TABLE 2:** Search strategy.

Search	Search details
Databases	Cochrane Online
	PubMed
	MEDLINE
	ScienceDirect
	Taylor & Francis Online
	CINAHL, eBook Collection, E-journals, ERIC, Health Source-Consumer Edition, Health Source-Nursing/Academic Edition, Humanities International Complete (via EBSCOhost)
Manual search	Google to obtain grey literature Citation search
Key words	‘Objective Structured Clinical Examination’ or ‘OSCE’ or ‘Health Science Education’ and ‘Quality’.

OSCE, Objective Structured Clinical Examination; CINAHL, Cumulative Index to Nursing and Allied Health Literature; ERIC, Education Resources Information Center.

The first and third authors independently screened titles and abstracts as well as the full text of obtained literature, according to the following criteria governing literature selection: research and non-research documents, as well as grey literature regarding the quality management of OSCE design in health science education, published in English, between January 2010 and March 2021.

Thirteen eligible full-text articles were critically appraised. The first author and an independent reviewer assessed each article using one of two Johns Hopkins appraisal tools: one for research evidence and one for non-research evidence. Each article received a score to determine its inclusion or exclusion in the data synthesis and extraction process. This score was calculated by dividing the number of ‘yes’ responses on the critical appraisal tool by the total number of items on the tool, and then multiplying by 100. As a result of the limited number of articles available on the topic and to ensure the inclusion of relevant articles with sufficient rigour, a minimum score of 60% was set as the threshold for article selection. The first and third authors independently extracted data from all 13 articles. Extracted data were synthesised using thematic analysis according to Cooper (1998). The extracted data were read, compared, ordered, and coded. The coded data were categorised and grouped under themes and sub-themes. A total of 12 quality measures to be adopted in quality OSCE planning and design were identified (see Table 3).

**TABLE 3:** Two sets of themes synthesi**s**ed into seven draft recommendations.

Themes from qualitative interviews and document analysis	Themes from integrative literature review	Synthesised data	Recommendations
**Theme One: Measures that facilitate quality in OSCE planning and design** 1.1 A peer review system 1.2 Control measures ensuring confidentiality 1.3 Pre-OSCE briefing and orientation 1.4 Validation of assessment tools	Quality measures to be adopted in the quality planning and design of OSCEs Establish an organising committee Conduct blueprinting and mapping of OSCE content Develop a bank of OSCE stations Select station writers	Themes 1.1, 1.2, 1.4, 2.1 & 2.4 (interviews & document analysis) Quality measure 1 (review)	Develop a policy framework and standard operating procedures as well as a stakeholder OSCE code of conduct
		Themes 1.1, 1.4, 2.1 & 2.3 (interviews & document analysis) Quality measure 2 (review)	Conduct blueprinting and mapping of the OSCE content
		Themes 1.4 & 2.1 (interviews & document analysis) Quality measures 1, 3, 4, 5, 6 (review)	Develop an OSCE station bank
		Themes 1.4 & 2.1 (interviews & document analysis) Quality measures 1, 7 & 8 (review)	Select a scoring rubric and standard-setting method
**Theme Two: Barriers that hinder quality in OSCE planning and design** 2.1 Inadequacy of OSCE assessment tools and documents 2.2 Physical space 2.3 Appropriate fidelity manikins 2.4 Sufficiently trained examiners	5. Select station types and decide on the number of stations 6. Conduct peer review workshops 7. Select a scoring rubric 8. Select a standard-setting method 9. Recruit and train examiners 10. Recruit and train standardised patients 11. Select an appropriate OSCE venue 12. Conduct an OSCE station piloting	Theme 2.4 (interviews & document analysis) Quality measures 1, 9 & 10 (review)	Recruit and train examiners and standardised patients*
		Theme 2.2 (interviews & document analysis) Quality measure 11 (review)	Select an appropriate OSCE venue
		Themes 1.3 & 1.4 (interviews & document analysis) Quality measures 1&12 (review)	Conduct an OSCE station piloting

*Source:* Adapted from Shah, R., Edgar, D.F. & Evans, B.J., 2018, ‘The use of simulated and standardised patients in education, training and assessment’, *Optometry in Practice* 19(1), 1

OSCE, Objective Structured Clinical Examination.

* , Standardised patients are individuals trained to simulate real patients’ medical conditions, histories, and symptoms consistently and accurately.

### Synthesising the qualitative data into draft recommendations

Synthesising the qualitative data into draft recommendations was carried out using data source triangulation. Data source triangulation refers to a combination of different sources (such as interviews and literature) wherein researchers synthesise both similarities and disparities to arrive at conclusions aligning with the findings according to Carter et al. (2014), as follows:

1. *Identifying the sets of themes* (two sets of themes were derived)
  - Set one: Three main themes with four sub-themes each from the qualitative interviews and document analysis related to measures that facilitate and barriers that hinder quality planning and design of OSCEs.
  - Set two: Twelve quality measures to be adopted in the quality planning and design of OSCEs from the integrative literature review.
2. *Comparing themes*: The authors read and noticed overlaps, intersections, or complementary aspects between the two sets of themes. For example, a peer-review system (theme 1.1 interviews and document analysis) was linked to conducting peer review workshops (Quality measure 6 – integrative literature review).
3. *Synthesising themes*: The authors integrated, refined, and categorised the themes from both sets to derive a unified understanding of the phenomenon under study. This involved looking for patterns, relationships, and connections between the themes. For example, while specific quality measures were crucial for the quality planning and design of OSCEs, it was the alignment of these quality measures with facilitating measures in place and addressing the barriers that enhanced the quality of OSCE planning and design particularly for the study context.
4. *Deriving and organising recommendations*: Once the integrated set of themes was established, the authors identified key insights and implications for practice or policy. These insights would form the basis of the recommendations. The authors organised the recommendations into a coherent framework, with a logic sequence enhancing the quality design of OSCEs. In this case, the recommendations were grouped according to overarching themes such as laying the foundation in terms of policy framework, standard operating procedures, and a code of conduct, followed by blueprinting and mapping, whereafter recommendations regarding the details of planning and designing the content of the OSCE were included, such as the OSCE bank, scoring rubrics, recruitment and training requirements, the OSCE venue and piloting. The recommendations largely followed the sequence of the identified quality measures from the integrative literature review as this was deemed logical in the planning and design of OSCEs, resulting in seven draft recommendations.

Table 3 outlines the two sets of themes, synthesised into seven draft recommendations.

### Review of the draft recommendations

The draft recommendations were independently reviewed via email by eight senior educators – of which seven held doctoral degrees and the eighth held a master’s degree – with expertise in OSCEs and guideline development using an adapted AGREE II (Appraisal of Guidelines for Research & Evaluation) tool (Brouwers et al. 2010). The AGREE II tool is a framework designed to assess the quality and rigour of clinical practice guidelines, consisting of 23 key items across six domains, namely, Scope and Purpose (overall aim, target population, specific health questions), Stakeholder Involvement (representation of intended users’ views), Rigour of Development (evidence synthesis and recommendation formulation), Clarity and Presentation (language and format), Applicability (implementation barriers, facilitators, and resource implications), and Editorial Independence (influence of funding and conflicts of interest), providing a standardised method to ensure guidelines are evidence-based, transparent, and applicable to clinical practice (Brouwers et al. 2010). Recommendation 3 was adjusted because of expert reviewer feedback, specifically with regard to the necessity for station writers who are educational experts with experience in health sciences and nursing education and for having between 10 and 20 OSCEs.

### Ethical considerations

Institutional ethical approval was granted by Nelson Mandela University’s Research Ethics Committee (Human [H-19-HEA-NUR-006]) on 08 July 2019. Before the study was commenced, consent was obtained from nurse educators, and applicable permission was obtained to assess external moderators’ reports.

## Results

### Recommendations

Table 4 outlines the final seven recommendations after review, including the level of evidence as per Lobiondo-Wood and Haber (2021). Lobiondo-Wood and Haber (2021) categorise levels of evidence into a hierarchy from the highest to lowest: Level I (systematic reviews or meta-analyses of Randomised Controlled Trials (RCTs) and evidence-based guidelines), Level II (well-designed RCTs), Level III (controlled trials without randomisation), Level IV (case-control or cohort studies), Level V (systematic reviews of descriptive and qualitative studies), Level VI (single descriptive or qualitative studies), and Level VII (expert opinions or committee reports), aiding in the assessment of the strength and reliability of evidence for clinical decision-making.

**TABLE 4:** Final recommendations after review.

Recommendations The following OSCE planning and design quality measures should be applied:	Source/data used to support recommendation	Level of evidence
1. Develop a policy framework and standard operating procedures as well as a stakeholder OSCE code of conduct	I, DA, ILR	IV and VII
2. Conduct blueprinting and mapping of the OSCE content	ILR	IV, VI and VII
3. Develop an OSCE station bank	I, DA, ILR	I, IV and VII
4. Select a scoring rubric and standard-setting method	ILR	I, IV, VI and VII
5. Recruit and train examiners and standardised patients	ILR	I, IV and VII
6. Select an appropriate OSCE venue	I, DA, ILR	IV and VII
7. Conduct an OSCE station piloting	I, DA, ILR	VII

Note: I (systematic reviews/meta-analysis randomised controlled trials/best practice guidelines); II (well-designed RCTs); III (controlled trials without randomisation); IV (single non-experimental studies); V (systematic reviews of descriptive studies); VI (single descriptive studies); VII (expert opinion articles).

OSCE, Objective Structured Clinical Examination; I, Interviews; DA, document analysis; ILR, integrative literature review.

The recommendations will now be described.

#### Recommendation One: Develop a policy framework and standard operating procedures as well as an Objective Structured Clinical Examination stakeholder code of conduct

***Rationale:*** Standardises OSCE designs, assists in preparing for a well-designed OSCE and provides guidance to OSCE stakeholders (students, nurse educators, external moderators) on what is acceptable behaviour and how to prevent and address unprofessional conduct and maintain discipline and OSCE credibility (Castro-Yuste et al. 2020; Khan et al. 2013).

Content experts (who may include internal staff and external moderators), with sufficient clinical, health sciences and nursing education experience and those acquainted with the curriculum, published principles and standards underlying OSCEs, that are appointed as part of the OSCE organising committee, must:

Develop an OSCE policy framework and standard operating procedures, including:

- The criteria for and establishment of an OSCE design committee
- Standards on the minimum resources required to conduct an OSCE (e.g. physical space, type of fidelity manikins, trained examiners)
- Moderation criteria
- Selection criteria of the final OSCE tools and documents and external examiners
- Stakeholder’s roles and responsibilities
- Criteria and procedures for the utilisation of the command system
- A system ensuring uniformity in terms of: OSCE start times, additional time per station and adjustments to OSCE tools across all campuses
- A system for the management of OSCE score variances and (in)eligibility for re-OSCEs
- Measures promoting correlations between formative and summative clinical assessments and ratifying and publishing results
- A system for the evaluation of OSCEs and student reflection and redress

Develop a code of conduct to address:

- Illicitly assisting students during OSCEs
- Unauthorised dissemination, sharing, or revelation of confidential OSCE-related information to students (referred to as ‘leaking’)
- Student, standardised patient, and examiner utilisation of electronic communication such as email, messages and video or audio recording devices during OSCEs
- That requires students, standardised patients, and examiners to sign a mandatory declaration regarding OSCE content confidentiality.

#### Recommendation Two: Conduct blueprinting and mapping of the Objective Structured Clinical Examination content

***Rationale:*** Ensures the examination of correct standards and appropriate skill, aligning directly with the students’ level of competence required at their level of training, and providing authentic clinical assessment opportunities, directly related to relevant aspects in delivering safe care and curriculum requirements (Hastie et al. 2014; Kelly et al. 2016; Mitchell et al. 2015; Obizoba 2018; Pell et al. 2010).

Content experts must:

- Align OSCE content with curriculum learning objectives to ensure that all components of the curriculum are proportionally assessed (Ware et al. 2014)
- Obtain consensus on sufficient OSCE stations that promote adequate content coverage aligned with students’ required clinical competences (Goh et al. 2016; Ogah et al. 2016)
- Use a prescribed method, tool, or instrument, selecting relevant OSCE station matter and ensure adequate inclusion of all learning domains and competences (Goh et al. 2016; Khan et al. 2013). This tool could include elements such as OSCE content per learning outcome, the number and duration of stations, station types for each clinical skill, weighting of OSCE content, instructions for students, examiners, and standardised patients as well as resource requirements such as manikins, standardised patients (Goh et al. 2016).
- Verify the OSCE content blueprinting tool through evaluation by three independent content experts to ensure relevance and objectivity, then have it signed off and handed over to the station writers (Goh et al. 2016).

#### Recommendation Three: Develop a bank of Objective Structured Clinical Examination stations

***Rationale:*** Improves OSCE efficiency, reliability and validity by: finding a suitable expert panel to design and write OSCE stations; providing an understanding regarding OSCE duration, resources required and strategies to be employed in an OSCE; and providing the opportunity for station writers to quality assure stations to confirm the clinical accurateness and suitability of the required clinical tasks by students (Khan et al. 2013).

Content experts should:

- Develop and maintain an OSCE station bank and related tools (Khan et al. 2013; Ware et al. 2014)
- Prior to incorporating an OSCE tool into the OSCE bank, it is recommended to undertake peer review, piloting, and psychometric analysis, as suggested by Khan et al. (2013)

Once OSCE blueprinting and mapping have been finalised, content experts:

- Should select station writers to design and develop stations using station writing templates
- Should select an educational expert to coordinate the writing of stations and guide station writers regarding the required station types for assessing selected curriculum outcomes (Hastie et al. 2014; Khan et al. 2013).
- To attain essential content representativity, a minimum of 10 and up to 20 stations, lasting between 5 and 10 min for each OSCE, should be prepared (Brannick, Erol-Korkmaz and Prewett 2011).
- Selecting a station type (e.g., unobserved, observed, linked and technology-enhanced stations) and station numbers should be based on the OSCEs’:
  ■ Aim, Capacity to evaluate domains of learning, Ability to maintain uniformity and standardisation in all stations regarding the equipment, stock, stationery, scoring rubrics and scenarios as well as the available resources and conditions of an institution (Agarwal et al. 2010).
- Peer review workshops are held for all station writers and nurse educators, enhancing capacity, to evaluate and critique (using a questionnaire to measure appropriateness and accuracy of the station content) and adapt stations written by their peers (Khan et al. 2013).

#### Recommendation Four: Select a scoring rubric and standard-setting method

***Rationale:*** Enables examiners to allocate scores based on the demonstrated clinical skills and the intended objective of the OSCE, fostering equitable outcomes in pass or fail evaluations (Daniels & Pugh 2018; Kamal et al. 2020; Khan et al. 2013).

Station writers, appointed as part of the OSCE organising committee, must:

- Choose a checklist when the aim of the OSCE is to evaluate the actions anticipated from students at each station (Hastie et al. 2014; Ware et al. 2014).
- Choose a holistic or global rating scale when the objective of the OSCE is to assess the execution of a specific action (Khan et al. 2013; Schleicher et al. 2017).
- Preferentially employ the criterion-referenced approach as a standard-setting method to ascertain the complexity and significance of each OSCE item and to establish threshold scores aligned with the necessary student proficiency (Hastie et al. 2014; Khan et al. 2013; Yousuf, Violato & Zuberi 2015).
- In the selection of the scoring, consider overriding factor as well as the assessment of the various domains including affective, cognitive and psychomotor skills.

#### Recommendation Five: Train recruited examiners and standardised patients

***Rationale***: It allows examiners to evaluate students consistently and objectively, while also helping standardised patients understand the significance of providing precise and dependable portrayals of the required clinical scenarios, repeatedly for each student participating in an OSCE (Gormley 2011).

In terms of training examiners – who will be assessors during the OSCEs and are sourced internally within the staff complement as well as externally from staff in nearby clinical facilities – prior to the OSCEs, content experts should:

- Develop an OSCE examiner training programme to:
  ■ train examiners according to minimum assessment standards
  ■ provide examiners the chance to practise scoring utilising appropriate scoring rubrics (Gormley 2011).
- Document the outcomes of the training workshops (Hastie et al. 2014).

In terms of training standardised patients, the OSCE organising committee:

- Should implement a robust training programme, which can include role play, equipment, and devices to simulate authentic medical interactions (Daniels & Pugh 2018).
- Following training, it is essential to assess the performance of each standardised patient before permitting them to participate in OSCEs (Khan et al. 2013).

#### Recommendation Six: Select an appropriate Objective Structured Clinical Examination venue

***Rationale:*** To have a dedicated, custom-built venue to run OSCEs in an appropriate way (Ware et al. 2014).

The OSCE organising committee should:

- Ensure that the venue – such as a custom-built skills laboratory – is mapped, considering the station placement and type (e.g. with patients, manned, unmanned) and flow patterns (Ware et al. 2014).
- Select the venue based on:
  ■ Its customisation to include essential resources such as high-fidelity patient simulators, task trainers, medical instruments, mock clinical setups, reference materials, anatomical models, simulation software, recording and playback systems, a standardised patient programme, assessment tools, IT support, scheduling systems, and inventory management to ensure comprehensive training and evaluation.
  ■ Available briefing, waiting and quarantine spaces, administrative offices, and refreshment zones.
  ■ A custom-built skills laboratory for OSCEs can include high-fidelity patient simulators, task trainers, medical instruments, mock clinical setups, reference materials, anatomical models, simulation software, recording and playback systems, a standardised patient programme, assessment tools, debriefing rooms, study and practice areas, IT support, scheduling systems, and inventory management to ensure comprehensive training and evaluation.
  ■ Capability to perceive the bells that signal the station rotation times during the OSCE (Ware et al. 2014)
  ■ In case recording technology is employed, a control room is required, overseeing live video feeds from every station (Ware et al. 2014).

#### Recommendation Seven: Conduct an Objective Structured Clinical Examination station piloting

***Rationale:*** To recognise and rectify practical discrepancies, ensuring impartiality and feasibility of assigned tasks within the recommended timeframe (Ware et al. 2014).

The OSCE organising committee should:

- Prior to finalising the stations into the OSCE, conduct station piloting. This involves familiarising examiners with the OSCE prerequisites and scoring criteria, along with evaluating the adequacy of instructions, the suitability of time allotment for each task, the coherence among tasks, and the arrangement of OSCE station sequence (Ware et al. 2014).

## Discussion

This study aimed to describe the development of evidence-based recommendations for strengthening the planning and design of OSCEs for a South African public College of Nursing. Seven recommendations were developed related to: (1) a policy framework, standard operating procedures and an OSCE stakeholder code of conduct; (2) blueprinting and mapping of the OSCE content; (3) a bank of OSCE stations, selection of station writers, types and numbers of stations, and conduct peer review workshops; (4) scoring rubric and standard-setting method selection; (5) examiners and standardised patients’ recruitment and training; (6) Objective Structured Clinical Examination venue selection; and (7) Objective Structured Clinical Examination station piloting. These recommendations can be implemented in the sequence they are presented, although some elements, such as the peer review workshop, could also include one or a series of workshops on the OSCE station bank and selecting a scoring rubric and standard-setting method (Recommendation Four) and training of recruited examiners and standardised patients (Recommendation Five).

Planning for and designing an OSCE requires the development of a policy framework, standard operating procedures, and codes of conduct as these lay the foundation for conducting OSCEs and can enhance and assure quality assessment in health education. To develop the documents, benchmarking of OSCEs can be performed (Saad et al. 2021). When creating the documents, it is crucial to consider alignment to Professional Board or Council requirements; the availability or required human, financial and material resources (equipment, venue) for the implementation of OSCEs; effective and efficient use of these resources (e.g. through psychometrics of OSCE banks); as well as to indicate when these documents should be reviewed to keep abreast with latest developments and requirements related to OSCEs (Hopwood, Myers & Sturrock 2021).

Blueprinting and mapping of the OSCE content, developing the OSCE bank and select a scoring rubric and standard-setting method should preferably be carried out by the same established OSCE design committee to ensure consistency, guided by the developed policy frameworks, operating procedures and code of conduct (Monti et al., 2020). Similar to developing the policy frameworks, standard operating procedures and code of conduct, for blueprinting and mapping the development of the OSCE bank and the selection of scoring rubrics and standard-setting methods should be aligned with the requirements of the Professional Council or Board, including the needed level of competence a nurse should have in accordance with their scope of practice, as recommended elsewhere (D’Aoust et al. 2022). Furthermore, a variety of blueprints, OSCE banks and scoring rubrics and standard-setting methods are required to have numerous test and assessment blueprints for a wide diversity of learning domains and test score validations (Raymond & Grande 2019).

When recruiting and training examiners, factors that have been reported to influence the examiner’s objectivity should be considered, including training, assessment expertise, and understanding of student and station types. Staff from one campus can be recruited and utilised as examiners at another campus, a process referred to as cross-examination. In addition, peers could be recruited as examiners to provide high-quality feedback on OSCEs but used with caution, depending on their familiarity with students and ability to conduct objective assessments (Sader et al. 2022). Considering the developed recommendations, training of recruited examiners and standardised patients, which can be performed through a series of workshops presented by OSCE committee members or external specialists, should preferably include information about OSCE policies, standard operating procedures, code of conduct, assessment processes and tools.

The venue should be considered during the development of the policy framework to ensure consistency in resources, as inconsistencies were identified as a problem in the study context. The venue should be booked in advance to avoid disruptions and to accommodate the number of bookings. Furthermore, the suitability of a venue is important in enhancing privacy and reducing noise, and therefore enhancing the reliability of an OSCE (e.g. students not being able to hear each other if performing a similar skill, affecting student outcomes) (Lim et al. 2023).

Finally, a mock OSCE could be used to pilot the OSCE and OSCE banks and tools, as this method has been reported to assist in students’ time management skills, alleviate student anxiety and improve their clinical skills. However, from the literature, a mock OSCE often seems to be used as a way of training students, examiners, and standardised patients (Gilani et al. 2022). A mock OSCE should, therefore, include a debriefing that not only focuses on students’ performance but also encourages self-reflection for students, standardised patients, and examiners involved in the process. Generally, little is known regarding piloting an OSCE, which requires further exploration.

In summary, the developed recommendations were well-supported by existing literature, except for the sixth recommendation related to selecting the appropriate OSCE venue. The developed recommendations can enhance the planning and design of OSCEs, vital in enhancing OSCE quality.

### Limitations

The study was limited for several reasons. Firstly, it focused on strengthening the OSCE planning and design as a basis for its implementation. Secondly, the recommendations were developed based on synthesised data from the College in South Africa and some recommendations may not apply to all educational contexts. Thirdly, external moderators’ first-hand accounts and opinions could not be obtained. Fourthly, limited evidence-based methodologies relating to non-clinical topics limited our ability to develop non-clinical recommendations such as this one. Lastly, utilising qualitative approaches for sampling and data collection limited the number of participants and thus limited generalisability.

## Conclusion

A set of seven evidence-based recommendations was formulated concerning the establishment of a policy framework, standard operating procedures, and a code of conduct for OSCE stakeholders. These recommendations also covered the blueprinting and arrangement of OSCE content and stations in the bank, as well as the selection of scoring rubrics and standard-setting approaches. In addition, it is advised to focus on examiner and standardised patient recruitment and training, venue selection for the OSCE, and the implementation of OSCE station piloting. These recommendations offer valuable guidance for nurse educators seeking to enhance the planning and design of OSCEs. This is pivotal in elevating the quality of OSCEs. However, the recommendations require further development, piloting, and implementation, considering alignment with national Professional Board and Council requirements, and factors that influence the objectivity of examiners. Finally, OSCE piloting should be further explored.
